# Engrams of Fast Learning

**DOI:** 10.3389/fncel.2020.575915

**Published:** 2020-10-28

**Authors:** Charlotte Piette, Jonathan Touboul, Laurent Venance

**Affiliations:** ^1^Center for Interdisciplinary Research in Biology, College de France, INSERM U1050, CNRS UMR7241, Université PSL, Paris, France; ^2^Department of Mathematics and Volen National Center for Complex Systems, Brandeis University, Waltham, MA, United States

**Keywords:** fast learning, one-shot learning (OSL), memory engram, synaptic plasticity (LTP/LTD), neuromodulation, neurocomputational models, artificial intelligence

## Abstract

Fast learning designates the behavioral and neuronal mechanisms underlying the acquisition of a long-term memory trace after a unique and brief experience. As such it is opposed to incremental, slower reinforcement or procedural learning requiring repetitive training. This learning process, found in most animal species, exists in a large spectrum of natural behaviors, such as one-shot associative, spatial, or perceptual learning, and is a core principle of human episodic memory. We review here the neuronal and synaptic long-term changes associated with fast learning in mammals and discuss some hypotheses related to their underlying mechanisms. We first describe the variety of behavioral paradigms used to test fast learning memories: those preferentially involve a single and brief (from few hundred milliseconds to few minutes) exposures to salient stimuli, sufficient to trigger a long-lasting memory trace and new adaptive responses. We then focus on neuronal activity patterns observed during fast learning and the emergence of long-term selective responses, before documenting the physiological correlates of fast learning. In the search for the engrams of fast learning, a growing body of evidence highlights long-term changes in gene expression, structural, intrinsic, and synaptic plasticities. Finally, we discuss the potential role of the sparse and bursting nature of neuronal activity observed during the fast learning, especially in the induction plasticity mechanisms leading to the rapid establishment of long-term synaptic modifications. We conclude with more theoretical perspectives on network dynamics that could enable fast learning, with an overview of some theoretical approaches in cognitive neuroscience and artificial intelligence.

## Fast Learning Behaviors

**Figure 1:**
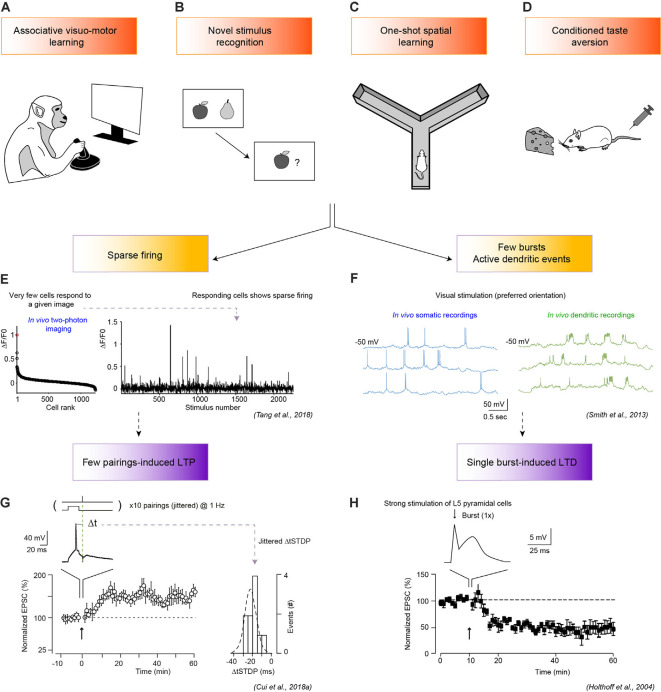
One-shot learning pathways to long-term synaptic plasticity. **(A–D)** Four examples of one-shot learning tasks: associative visuomotor learning **(A)**, recognition of a novel stimulus **(B)**, one-shot spatial learning in a maze **(C)**, and conditioned taste aversion (CTA; **D**). **(E,F)** Associated spike patterns include sparse firing **(E)** or a few bursts **(F)**. **(E)** Sparse firing (right panel) is illustrated by two-photon imaging recording of the primary visual cortex (V1) of an awake and behaving monkey during associative visuomotor learning (fixation task). Very few V1 neurons respond strongly to the natural visual stimuli considered (left panel), with a sharp peak in the rank-ordered distribution of population calcium-responses. Adapted from Tang et al. (2018). **(F)** Somatic, and dendritic patch-clamp whole-cell *in vivo* recordings in pyramidal cells of the visual cortex in mice show the emergence of few orientation-tuned somatic bursts and active dendritic events during the presentation of square-wave grating visual stimuli. Adapted from Smith et al. (2013). **(G)** With a spike-timing-dependent plasticity (STDP) paradigm, few spike pairs induce eCB-dependent long-term potentiation (LTP) at cortico-striatal synapses in rat brain slices. Top: protocol: 10 post-pre pairings of cortical stimulation and striatal spike, with a delay between pre- and postsynaptic activity centered at Δ*t* (−20 ms) with jitter to mimic noisy *in vivo* inputs, lower-right panel (std σ_Δt_ = 6.4 ms). The lower left panel shows the progressive establishment of LTP (*n* = 9 cells). Adapted from Cui et al. (2018b). **(H)** A single burst evokes a long-term depression (LTD) at layer 5 V1 pyramidal neuron in a rat brain slice. Top panel, somatic current recording upon single burst stimulation with the second peak reflecting a local dendritic spike. The bottom panel, averaged time-courses of LTD induced by a single burst (*n* = 8 cells). Adapted from Holthoff et al. (2004). In **(H,G)** arrows indicate the stimulation protocol. Permissions for the copyright of the adapted Figures in panels **(E–H)** have been obtained.

Fast learning mechanisms are best characterized in one-shot or single-trial learning paradigms which lead to memory formation after a single and brief (few hundred milliseconds to few minutes) exposure to relevant stimuli (Figures 1A–D). Indeed, what distinguishes fast learning are the features of the encoding stage (or learning experience): *fast learning* refers here to situations where memory traces are rapidly formed without requiring repetitions of the learning experience.

In animal models, a single exploration of a new place or novel objects can evoke long-lasting memories, as assessed in rodents using the delayed-matching-to-place task in a watermaze or novel object recognition (Ennaceur and Delacour, 1988; Steele and Morris, 1999; Nakazawa et al., 2003; Clarke et al., 2010). Paradigms using positive or negative reinforcement are also used to boost one-shot associative learning. Examples from rodents include odor-reward associations (Roullet et al., 1997; Armstrong et al., 2006), fear conditioning, and inhibitory avoidance tasks (Venable and Kelly, 1990; Izquierdo et al., 1997; Sacchetti et al., 2002; Vale et al., 2017) or conditioned taste aversion (CTA; Escobar et al., 2002). Positively reinforced one-shot learning of multiple visuomotor associations has also been reported in pigeons and baboons (Cook and Fagot, 2009).

In humans, episodic memory, referring to the formation and maintenance of memory traces of unique experiences, illustrates well fast learning. It should be noted that the existence of episodic memory in non-human mammals has remained controversial in the literature (Clayton et al., 2001; Fellini and Morellini, 2013). Behavioral tests in humans have focused on the familiarity and recollection components of episodic memory, using respectively forced recognition tasks (Standing et al., 1970; Rutishauser et al., 2006) and source memory tests (Jacoby, 1991; Harlow and Donaldson, 2013). One-shot associative learning can be used to test additional aspects of episodic memory, such as objects-place associations (Ison et al., 2015; Brodt et al., 2018). One-shot associative learning can also extend to more abstract representations and semantic memories, such as in learning new words (Carey and Bartlett, 1978), concepts, or object categories (Biederman, 1987).

## Neuronal Activity During Fast Learning

*In vivo* recordings of spiking activity during a fast learning experience reveal sparse activity modulation and the occurrence of few bursting events (Figures 1E,F). During a ~1 s visual stimulation, few neurons from human or monkey anterior and medial temporal cortical lobes and basolateral amygdala (BLA) fire ~5–20 spikes (Messinger et al., 2001; Rutishauser et al., 2006; Ison et al., 2015). These brief presentations of a novel stimulation can however induce significant changes in neuronal activity lasting for at least 10 h (Fried et al., 1997; Xiang and Brown, 1998; Rutishauser et al., 2006) and even allow distinguishing between subjects having perceived the stimulus as a novel or familiar (Fried et al., 1997; Rutishauser et al., 2006). During active touch or passive sensing, sparse activity is also detected in the visual, somatosensory, and auditory cortices, with 0.5–5% of neurons increasing their firing rates in cats, ferrets, or rodents (Yao et al., 2007; Hromádka et al., 2008; Jadhav et al., 2009; O’Connor et al., 2010; Tang et al., 2018; Yoshida and Ohki, 2020; Figure 1E). Such sparse activity is accompanied by the emission of bursts in pyramidal cells, coupled to active dendritic events, in sensory cortices and in the hippocampus (Xu et al., 2012; Smith et al., 2013; Takahashi et al., 2016; Manita et al., 2017; Figure 1F). Moreover, a single presentation of a sensory stimulus induces short-term reverberatory patterns in spontaneous activity during at least a few minutes (Yao et al., 2007), and persistent changes in receptive fields, lasting for several hours (Fritz et al., 2003). Similarly, a single passage in a maze, inducing few bursting events (O’Keefe and Recce, 1993; Epsztein et al., 2011), leads to new stable place fields (Mehta et al., 2000; Bittner et al., 2017). After visuo- and odor-motor association fast learning tasks, long-lasting task-related responses emerge rapidly in the prefrontal cortex and striatum (Pasupathy and Miller, 2005; Cromer et al., 2011) and in the striatal olfactory tubercle (Millman and Murthy, 2020). Similar phenomena are observed in the auditory cortex during the very first fear conditioning trials (Edeline et al., 1993) or in the BLA-projecting neurons of the gustatory cortex after CTA (Lavi et al., 2018).

Altogether, despite the low number of spikes or bursting events, the induction of persistent selective responses can be initiated after a single or few stimulus presentations across the brain and thus serve as neural indicators of acquired memory traces.

## Fast Learning-Induced Neuronal Long-Term Changes

Long-term changes have been reported subsequently to various fast learning tasks. In particular, some studies identified such long-term changes in cells activated during the learning experience, thus giving privileged access to understanding the nature of fast learning engrams, that are the set of “enduring physical and/or chemical changes elicited by learning and that underlie a newly formed memory” (Josselyn et al., 2015; Tonegawa et al., 2015; Josselyn and Tonegawa, 2020).

### Fast Learning-Induced Structural, Synaptic, and Intrinsic Plasticity Changes

One-shot learning tasks can be sufficient to activate some immediate-early genes or cellular transcription factors in hippocampal, BLA, or cortical neurons (Radulovic et al., 1998; Sananbenesi et al., 2002; Miyashita et al., 2009; Liu et al., 2012; Fellini and Morellini, 2013; Tayler et al., 2013). Such markers, in conjunction with genetic manipulations, have been used to identify engram cells in fast learning tasks, by tracing active cells during memory encoding and reactivated cells during retrieval (Tayler et al., 2013; Denny et al., 2014; DeNardo et al., 2019), or by manipulating their activity (Han et al., 2009; Choi et al., 2011; Liu et al., 2012; Josselyn and Tonegawa, 2020).

Long-term structural and synaptic plasticity changes have been reported in subcortical areas after a fast learning experience and specifically linked to engram cells in fear conditioning protocols: *in vivo* measurements of field-EPSPs reveal long-term potentiation (LTP) in rat dentate gyrus during spatial exploration, and at CA3-CA1 synapses after novel object recognition or an inhibitory avoidance task in mice (Moser et al., 1993; Whitlock et al., 2006; Clarke et al., 2010). Pharmacological manipulations and *ex vivo* electrophysiology support these findings, through the observation of synaptic plasticity occlusion or the facilitation of plasticity induction under subthreshold stimulation protocols (Sacchetti et al., 2002; Nakazawa et al., 2003; Whitlock et al., 2006; Romberg et al., 2013). Furthermore, a single contextual fear conditioning induces a structural and functional potentiation between CA3 and CA1 engram cells, with increased spine size and number, and *ex vivo* LTP expression (Choi et al., 2018), as well as a presynaptic LTP between cortical inputs and BLA engram cells (Nonaka et al., 2014).

Long-term intrinsic plasticity changes are also evoked by a one-shot learning experience: hippocampal neurons activated by a single fear conditioning protocol become more excitable during several days, thus potentially facilitating subsequent learning (Crestani et al., 2018), and changes in membrane excitability correlate with freezing levels and can be reversed after a single extinction trial (McKay et al., 2009). Interestingly, the magnitude of the increase in membrane excitability is comparable to those reported after repeated trials of conditioning protocols (Moyer et al., 1996; Saar et al., 1998; Song et al., 2012; Sehgal et al., 2014).

### Fast Learning Neocortical Engrams

While fast learning mechanisms have historically been associated to allocortical and subcortical areas (McClelland et al., 1995; Buschman and Miller, 2014), rapid formation of neocortical engrams have been uncovered in several fast learning tasks (Hofstetter et al., 2016; Kitamura et al., 2017; Brodt et al., 2018; Hebscher et al., 2019). First, the formation of neocortical engrams rapidly occurs in parallel to subcortical and allocortical changes and contributes to early system consolidation mechanisms: after a contextual fear conditioning, prefrontal neurons immediately engage in the formation of memory engrams, that progressively become functional with a maturation process requiring hippocampal and BLA inputs, and serve as long-lasting memory traces while hippocampal engram cells become silent during remote memory retrieval (Tayler et al., 2013; Kitamura et al., 2017; Matos et al., 2019). Furthermore, synaptic changes rapidly emerge in cortical-dependent one-shot learning tasks, such as after new word learning in human cortical areas involved in language and reading (Hofstetter et al., 2016), or in the perirhinal cortex during recognition memory (Brown and Banks, 2015). Also, after CTA, long-term actin rearrangements occur in gustatory and prelimbic cortices (Bi et al., 2010), and *N*-methyl-D-aspartate receptor (NMDAR)-mediated LTP at BLA-cortical synapses is occluded *in vivo* for at least 5 days (Escobar et al., 2002; Rodríguez-Durán et al., 2011).

These experiments suggest that the neural correlates of one-shot learning experience engage molecular machinery and cellular processes similar to those reported after repetitive training, such as the long-term activation of the same genetic markers, the establishment of long-term synaptic and structural changes, or the requirement for NMDA receptors. Yet, several questions remain to be elucidated, such as the modalities of induction and the extent (both in terms of magnitude and number of cells engaged) of fast learning-induced changes compared with repetitive training.

## Deconstructing Fast Learning Synaptic Plasticity Mechanisms

In light of the aforementioned results and given the neuronal activity patterns observed during fast learning, we now present some hypotheses on the induction mechanisms of fast learning-induced synaptic changes. Two natural hypotheses ensue: fast learning could constitute a condensed version of synaptic events similar to those occurring during repetitive learning and/or fast learning could be enabled by intrinsically stronger synaptic events. In the former scenario, the difference between the two learning processes would lie in the sensitivity of plasticity induction, a factor that could be modulated by the initial state of the synapses (with for instance more or less available membrane voltage-gated channels) or the efficiency of neuromodulatory systems. In the latter scenario, fewer synaptic events may be needed to initiate long-term changes, such that a one-shot exposure may be sufficient to drive the formation of a memory trace. The excitability of the active cells and/or the activation of some specific membrane channels could promote the generation of larger events, such as amplificatory dendritic phenomena or larger post-synaptic responses leading to stronger calcium influx.

**Figure 2:**
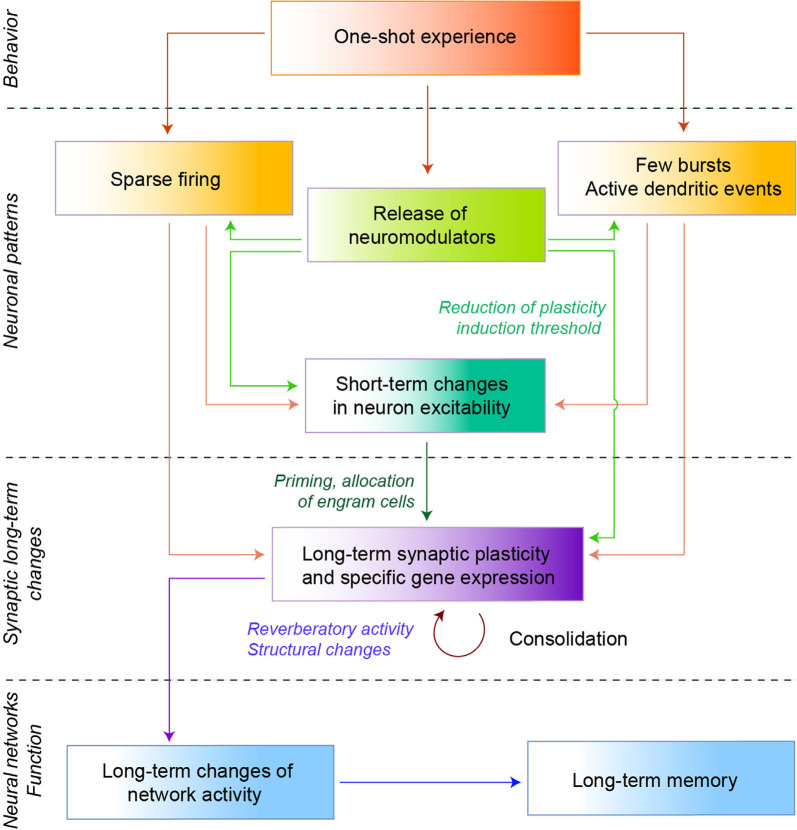
Elementary cellular mechanisms of fast learning. Schematic diagram of the putative cellular mechanisms leading from a one-shot experience to a long term memory. A one-shot experience leads a small fraction of cells to fire a few spikes (sparse network activity, left), and/or few bursts accompanied by active dendritic events (right). Neuromodulators can also be released, particularly in the presence of salient elements in the stimulus (e.g., novelty or rewards). *In vitro* evidence showed that neuromodulators play a role in the selection of patterns, as well as in the induction of short-term changes in excitability that could prime neurons to become engram cells. Neuromodulators can also lower the synaptic plasticity induction threshold, thereby facilitating long-term plasticity. Such long-term plasticity can then be consolidated by specific gene expression, structural changes, or reverberatory activity, altogether leading to the emergence of long-term memory following a single experience.

Due to the sparsity of direct links between neuronal activity and synaptic changes of engram cells, we mostly refer here to *in vitro* electrophysiological experiments using brief stimulation protocols, mimicking activity patterns observed during a fast learning experience, and capable of inducing long-term synaptic plasticity in randomly selected neurons (Figures 1G,H, 2). We also examine the impact of additional factors such as short-term intrinsic changes and neuromodulators. The following experiments provide first insights into the putative induction mechanisms, but further work is needed to apply similar protocols on to-be engram cells and link their artificially-induced long-term changes to learning, as well as to observe *in vivo* natural plasticity rules.

### Induction of Long-Term Synaptic Plasticity Under Sparse and Burst Firing

#### Sparse Firing

Only a few spikes may be transmitted between neurons during a one-shot experience: this constraint could potentially conflict with the classic Hebbian framework requiring repetition or persistence of a given activity pattern to induce stable long-term synaptic plasticity (Hebb, 1949). Yet, *in vitro* studies demonstrate that few coincident activities can be sufficient to induce spike-timing-dependent plasticity (STDP) in several brain areas. No more than ~10–15 spike-EPSP pairings between L2/3 pyramidal cells of the visual cortex are sufficient to induce Hebbian LTP, while LTD induction requires ~30 spikes (Froemke et al., 2006), with classical induction STDP protocols relying on 75–150 pairings. Interestingly, LTP magnitude is not affected by adding more pairings, suggesting a potentially rapid memory acquisition through the induction of an abrupt all-or-none LTP in response to minimal stimulation, as observed at CA3-CA1 synapses (Petersen et al., 1998). Moreover, in striatal projecting neurons and in L5 pyramidal cells of the somatosensory cortex, *in vitro* STDP paradigms involving ~5–15 cortico-striatal pairings induce an endocannabinoid-mediated LTP (eCB-LTP; Cui et al., 2015, 2016, 2018a; Xu et al., 2018; Gangarossa et al., 2020; Figure 1G). This LTP disappears for 25–50 pairings, only to re-emerge at 75 pairings as an NMDAR-mediated LTP (NMDAR-LTP). Importantly, eCB-LTP is more robust to spike jittering compared to NMDAR-LTP, and can thus arise in a noisy environment (Cui et al., 2018b; Figure 1G). Such a phenomenon could be critical to fast learning since the reliability of spiking activity in response to sensory stimuli might not yet be fully established (Yao et al., 2007). Interestingly, a recent study establishes that transient (10 s to 5 min) neuronal activation as well as a 1-min presentation of flashing visual stimuli specifically activate the MAPK/ERK signaling, and are sufficient to induce the first wave of primary response genes (Tyssowski et al., 2018).

#### Burst and Active Dendritic Events

Since bursts are transmitted more efficiently than isolated spikes, they could increase the signal-to-noise ratio of the network information transmission, and therefore appear as a privileged signal for inducing long-term changes during fast learning (Lisman, 1997; Krahe and Gabbiani, 2004; Figure 2). Indeed, triggering a single synaptic stimulation of L5 pyramidal neurons of the visual cortex induces LTD *in vitro*, only if the paired excitatory postsynaptic potential (EPSP) produces an NMDAR-dependent dendritic spike (Holthoff et al., 2004; Figure 1H). Similarly, a single burst of activity in Schaffer collaterals induces LTP, under the condition of triggering a postsynaptic dendritic spike and activating NMDAR and L-type voltage-gated calcium channels in CA1 pyramidal cells (Pike et al., 1999; Wittenberg and Wang, 2006; Remy and Spruston, 2007). Importantly, a pioneer *in vivo* study by Bittner et al. (2017) shows that during spatial navigation, spatially tuned entorhinal and CA3 inputs arriving 2–3 s before or after a CA1 post-synaptic calcium plateau potential can induce NMDAR-LTP and new place fields in CA1 pyramidal cells, thus paving the way for behavioral time-scale plasticity. These findings, also replicated *in vitro* (Bittner et al., 2017), bridge the gap between the timescales of behavioral learning and synaptic changes, and offer an alternative mechanism to a purely Hebbian framework that may be at stake in episodic-like memories.

### Factors Facilitating Long-Term Synaptic Plasticity Under Sparse and Burst Firing

#### Intrinsic Plasticity

Changes in neuronal excitability not only support synaptic changes as described above but can also act as a short-term priming mechanism (Figure 2). Indeed, intrinsic modulation of neuronal excitability generally has a lower induction threshold than synaptic plasticity and could contribute to induce early changes in neuronal activity (Titley et al., 2017) that will later favor the establishment of synaptic plasticity, even under sparse activity (Sah and Bekkers, 1996; Louise Faber et al., 2005). In this sense, artificially elevating neuron excitability selectively *in vivo* reveals place-cell activity in previously silent neurons (Lee et al., 2012) and turns these neurons into engram cells during a fear memory paradigm (Yiu et al., 2014). Interestingly, neurons of the piriform cortex are initially more excitable in fast learners compared to slow learners, with differences disappearing as their performance on the odor-discrimination task converges upon stimulus repetitions (Cohen-Matsliah et al., 2009).

#### Neuromodulation

Exposure to novel or salient stimuli releases neuromodulators necessary for spatial memory, context, and object recognition or one-shot emotional learning (Duszkiewicz et al., 2019; Likhtik and Johansen, 2019). Importantly, neuromodulators can favor the occurrence of specific synaptic events and neuronal activity patterns as well as lower the threshold for inducing long-term synaptic changes (Figure 2). Acetylcholine amplifies cue encoding at sensory cortical sites through inhibition, which allows the emission of larger bursts in presence of meaningful stimuli (Froemke et al., 2007; Letzkus et al., 2011). Also, under the presence of a cholinergic agonist, a single burst at the peak or trough of theta-rhythms induces *in vitro* LTP or depotentiation at CA3-CA1 synapses respectively (Huerta and Lisman, 1995). In the same vein, single burst-mediated LTP is more efficient with GABAergic transmission antagonists, suggesting that the excitation/inhibition balance, partly under neuromodulatory control, shapes the induction of fast learning plasticity (Remy and Spruston, 2007). Finally, dopamine expands the time window for NMDAR-LTP induction at CA3-CA1 synapses *in vitro* and decreases the threshold number of spike pairs (from 100 to 5–10) required for LTP (Zhang et al., 2009).

#### Reverberating Activity, as a Bypass to Sparse Firing

In addition to plasticity rules activated by small numbers of spikes, spontaneous replications of the associated neuronal activity may contribute to consolidating one-shot memories according to the classical Hebbian framework (Figure 2). Reverberating activity is observed especially during slow-wave sleep up to 48 h after a transient tactile exploration of novel objects (Ribeiro et al., 2004), and replay of past trajectories, in awake or asleep animals, is associated to memory consolidation in the hippocampus, ventral striatum and neocortex (Wilson and McNaughton, 1994; Hoffman and McNaughton, 2002; Pennartz et al., 2004; Ólafsdóttir et al., 2018). Interestingly, the awake replay is more prevalent and precise for trajectories in novel environments or associated with salient elements (e.g., a reward, Carr et al., 2011) and could thus particularly reinforce memory traces of unique salient experience.

## Reconstructing Fast Learning in Neuronal Networks

### Fast Learning and Slow-Fast Network Dynamics

If fast learning is best epitomized in one- or very few-shots learning tasks, it may also apply to the initial stages of repetitive and sustained training (Karni et al., 1998; Muellbacher et al., 2002; Costa et al., 2004; Qu et al., 2010; Law et al., 2014). As such, cortical engrams emerge in one-shot learning tasks as described above, but also in the initial phases of procedural learning (Karni et al., 1998; Muellbacher et al., 2002; Qu et al., 2010). Importantly, cortical representations acquired slowly over time are also essential in guiding future fast learning, which usually relies on prior knowledge and existing schemata such as when learning associations between known elements or novel words (Tse et al., 2007, 2011; Hebscher et al., 2019). Thus, one should keep in mind that fast and slow cortical dynamics are closely intertwined as exemplified in tasks necessitating high levels of cognitive flexibility (Pasupathy and Miller, 2005; Tse et al., 2007; Durstewitz et al., 2010; Siniscalchi et al., 2016; Perich et al., 2018; Remington et al., 2018; Rikhye et al., 2018).

### How to Control Learning Speed?

If fast learning can confer strong advantages, e.g., in survival-threatening situations, this strategy is not always adapted and can potentially lead to detrimental responses, such as superstitious behaviors, when an outcome is too rapidly associated with the wrong cause. Typically, procedural learning based on trial-and-error, or reinforcement learning, in which a trade-off slowly balances exploration and exploitation, necessitates several sessions before reaching an optimal behavior. Hence, control of the learning speed or meta-learning should also be seen as a critical component of the learning process. Yet, little is known about how the brain is implementing and switching between different learning strategies. Recent studies highlight how an accurate tracking of feedback (Mao et al., 2019) or stimulus saliency (Ceballo et al., 2019), amplified by cortical ensembles, parallels with learning speed. The degree of uncertainty of stimulus-outcome associations also regulates the learning speed in humans and controls the coupling strength between the hippocampus and ventrolateral prefrontal cortex (Lee et al., 2015).

### A Computational Perspective on a Fast Learning

Physiologically realistic models of fast learning have remained scarce and may require the development of new synaptic learning paradigms (Brea and Gerstner, 2016). Yet, phenomenological models of fast learning and machine learning algorithms have proposed various mechanisms providing a guide for the development of physiological models and offer testable hypotheses for experimentalists. Fast learning is affected with particular severity by two critical difficulties and exacerbates Hebb’s *sensitivity-stability* dilemma (Hebb, 1949): (i) small sample sizes hamper generalization capabilities and pattern inference; and (ii) *catastrophic interference*, the abrupt forgetting of previously learned information, can be easily encountered due to the high plasticity rates required by fast learning (McCloskey and Cohen, 1989; Ratcliff, 1990). To overcome these difficulties, various theories have been proposed. Synapses with hierarchical plasticity levels (Fusi et al., 2005) or selective consolidation of synapses (Leimer et al., 2019) allow fast acquisition and better retention of memories. In cognitive neurosciences, three key principles stand out as crucial for fast learning (Lake et al., 2017): the ability to compose simple primitives (and therefore use prior learning), identify causalities, and meta-learning (i.e., learning to learn). Combined in a generative hierarchical Bayesian framework, these mechanisms allowed the acquisition of new concepts after a single presentation, with performance comparable to human best one-shot learners (Lake et al., 2011, 2015).

In machine learning, various methods were developed to learn from few examples (Botvinick et al., 2019; Wang et al., 2020), with applications to an inference of situations with intrinsically limited samples (Shu et al., 2018) or for the prediction of rare events (Kaiser et al., 2017). In particular, *data augmentation* methods consist in adding to the dataset copies of the original data, modified according to specific rules, such as imposing invariances (Perez and Wang, 2017). Such repetitions of slightly modified stimuli could be akin to a sort of reverberating activity or replays. *Concept* *(transfer, or multitask)* *learning* consists in using and combining prior knowledge, potentially acquired from learning other tasks. These techniques integrate task-specific fast-learning modules and generic modules that learn from various tasks on generally slower timescales. These generic modules either *transfer* to the fast learning task a generic skill needed (Pan and Yang, 2009) or guide the task in the process of learning (Schaul and Schmidhuber, 2010; Finn et al., 2017). This typically echoes the aforementioned slow-fast plasticity interactions between brain areas. Recently, computational models of meta-learning have shown how a natural division of labor into functionally specialized clusters may arise in the prefrontal cortex, thus making it possible to compose multiple task elements for rapidly learning new tasks (Wang et al., 2018; Yang et al., 2019). Finally, adding *external memories* (Graves et al., 2014; Weston et al., 2014), storing past experiences (if not all, the most recent or most salient) allows few-shot learning and can further be combined with meta-learning algorithms to increase learning efficiency (Santoro et al., 2016; Ritter et al., 2018).

## Conclusion

Fast learning is thus a crucial component in daily life memory acquisition that involves one-shot learning experiences. If fast learning can be characterized by the brevity and rarity (or even uniqueness) of the learning experience, the processes involved in memory acquisition and maintenance are embedded in multiple timescales, considering their interactions with meta-learning and consolidation systems. While current findings have lifted part of the veil on fast learning engrams, several mechanisms remain to be further elucidated. In particular, causal interactions between minimal activity patterns and the induction of long-term changes during one-shot learning task remain to be further explored *in vivo*, as well as the cellular and molecular determinants controlling the learning speed across brain areas and in different contexts. These elucidations could uncover additional components of cellular and synaptic-based learning rules and would allow the development of more physiological models of fast learning.

## Author Contributions

CP wrote the “Fast Learning Behaviors, Neuronal Activity During Fast Learning, Fast Learning-Induced Neuronal Long-term Changes, Deconstructing Fast Learning Synaptic Plasticity Mechanisms, and Reconstructing Fast Learning in Neuronal Networks” sections and designed (Figures 1, 2). JT wrote the “A Computational Perspective on a Fast Learning” section and designed Figure 2. LV edited all versions of the manuscript and designed the Figures 1, 2. All authors contributed to the article and approved the submitted version.

## Conflict of Interest

The authors declare that the research was conducted in the absence of any commercial or financial relationships that could be construed as a potential conflict of interest.

## Abbreviations

BLA: basolateral amygdala
CTA: conditioned taste aversion
eCB: endocannabinoid
EPSP: excitatory postsynaptic potential
LTD: long-term depression
LTP: long-term potentiation
mPFC: medial prefrontal cortex
NMDAR: N-methyl-D-aspartate receptor
NMDAR-LTP: NMDAR-mediated LTP
STDP: spike-timing-dependent plasticity.
